# Antimycobacterial Activities of *N*-Substituted-Glycinyl 1*H*-1,2,3-Triazolyl Oxazolidinones and Analytical Method Development and Validation for a Representative Compound

**DOI:** 10.3390/scipharm85040034

**Published:** 2017-10-02

**Authors:** Naser F. Al-Tannak, Oludotun A. Phillips

**Affiliations:** Department of Pharmaceutical Chemistry, Faculty of Pharmacy, Kuwait University, P.O. Box 24923, Safat 13110, Kuwait; dphillips@hsc.edu.kw

**Keywords:** antimycobacterial activity, linezolid, PH-189, substituted-glycinyl triazolyl-oxazolidinone, quadrupole-time of flight mass spectrometry, ultra High Performance Liquid Chromatography

## Abstract

Twelve *N*-substituted-glycinyl triazolyl oxazolidinone derivatives were screened for antimycobacterial activity against susceptible (*Mycobacterium* *tuberculosis* (*Mtb*) H37Rv) and resistant (isoniazid (INH)-resistant *Mtb* (SRI 1369), rifampin (RMP)-resistant *Mtb* (SRI 1367), and ofloxacin (OFX)-resistant *Mtb* (SRI 4000)) *Mtb* strains. Most of the compounds showed moderate to strong antimycobacterial activity against all strains tested, with minimum inhibitory concentration (MIC) value ranges of 0.5–11.5, 0.056–11.6, 0.11–5.8, and 0.03–11.6 μM, and percent inhibition ranges of 41–79%, 51–72%, 50–75%, and 52–71% against *Mtb* H37Rv, INH-R, RMP-R, and OFX-R *M.* *tuberculosis*, respectively. The 3,5-dinitrobenzoyl and 5-nitrofuroyl derivatives demonstrated strong antimycobacterial activities with the *N*-(5-nitrofuroyl) derivatives (**PH-145** and **PH-189**) being the most potent, with MIC value range of 0.3–0.6 μM against all strains tested. Compounds were not bactericidal, but showed intracellular (macrophage) antimycobacterial activity. A reliable validated analytical method was developed for a representative compound **PH-189** using Waters Acquity ultra High-Performance Liquid Chromatography (UHPLC) system with quaternary Solvent Manager (H-Class). A simple extraction method indicated that **PH-189** was stable in human plasma after 90 min at 37 °C with more than 90% successfully recovered. Moreover, stress stability studies were performed and degradants were identified by using UHPLC-ESI-QToF under acidic, basic, and oxidative simulated conditions.

## 1. Introduction

**Figure 1:**
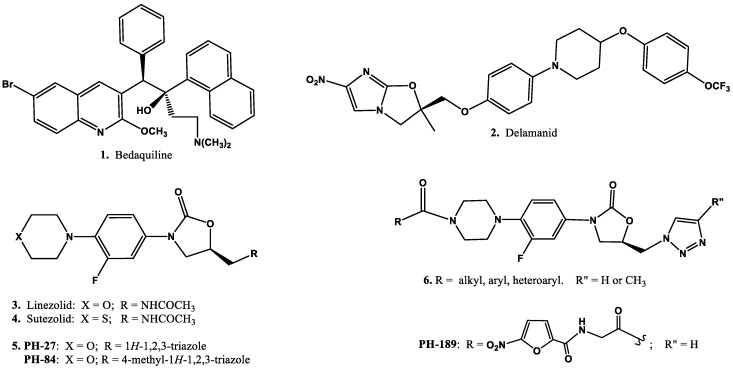
Chemical structures of anti-tubercular agents and oxazolidinone derivatives.

Tuberculosis (TB) is a highly contagious and fatal disease caused by *Mycobacterium tuberculosis* (*Mtb*). TB ranks among the top 10 causes of death worldwide, and is a major threat to the global healthcare system. The World Health Organization (WHO) reported that about 10.4 million people fell ill with TB with approximately 1.8 million deaths, including 0.4 million deaths among human immunodeficiency virus HIV infected patients and over 95% of TB deaths occurring in low- and middle-income countries [1]. The emerging incidences of multidrug-resistant TB (MDR-TB), extensively drug-resistant TB (XDR-TB) and more recently totally drug-resistant tuberculosis (TDR-TB) continue to impact the clinical outcomes of TB significantly making treatment more difficult to attain [1,2]. TDR-TB commonly refers to tuberculosis caused by *Mtb* strains that are resistant to all available first-line as well as second-line TB drugs. The development of resistance to first-line and second-line TB drugs coupled with the fact that current clinical treatment regimens for drug-resistant tuberculosis, MDR-TB, and XDR-TB are costly, complex, of lengthy duration, and associated with severe and life-threatening side effects which continue to impact clinical outcomes significantly. Therefore, there is an urgent need for the development of newer anti-TB agents [3,4]. Newer TB drugs should possess many properties, including activity against both replicating and non-replicating *M. tuberculosis*, they should act via a new target or mode of action against MDR-TB, XDR-TB, and TDR-TB, they should exhibit no antagonism to other TB drugs, and should be compatible with most HIV drugs since many TB patients are also co-infected with HIV [2]. Recently, two new TB drugs—bedaquiline, a diarylquinoline (**1**, Figure 1), and delamanid, a nitroimidoxazole (**2**, Figure 1)—have received conditional stringent regulatory approval and have World Health Organization interim policy guidance for their use in MDR-TB [2,4,5], adding to the anti-TB armamentarium. Both drugs have a unique mechanism of action, but are not totally devoid of serious adverse effects. For example, bedaquiline has been associated with induced arrhythmias and hepatotoxicity, and hence may be avoided in patients with cardiac problems [6]. 

Oxazolidinones, exemplified by linezolid (**3**, Figure 1) and sutezolid (**4**, Figure 1), represent a relatively new class of antibacterial agents with potent activity against Gram-positive bacterial pathogens, including multidrug-resistant strains—namely, methicillin-resistant *Staphylococcus aureus* (MRSA), penicillin-resistant *S. pneumoniae* (PRSP), vancomycin-resistant enterococci (VRE) [7] and MDR-TB and XDR-TB [2,8,9,10]. This class of compounds inhibit bacterial protein biosynthesis by binding to sites on the bacterial ribosomes, thus preventing formation of the functional 70S initiation complex [11]. 

Structural modification efforts around the phenyl-oxazolidinone moiety from our laboratory identified novel morpholino- and *N*-substituted-piperazino-oxazolidinone derivatives (**5** and **6**, Figure 1) as potent antibacterial agents active against susceptible and resistant Gram-positive bacterial strains, including *M. tuberculosis* [12,13,14,15]. Moreover, based on the potent antibacterial activity of the *N*-substituted-glycinyl piperizino oxazolidinone derivatives against Gram-positive bacterial strains [12], we decided to evaluate the antimycobacterial activity of selected examples in this class. Furthermore, because of the excellent in vitro antimycobacterial activity of the selected derivatives and proposed future plans to perform in vivo studies to investigate the antimycobacterial effect of selected examples on animal model, we decided to develop a rapid analytical method for a representative member of this class—namely, *N*-(5-nitrofuran-2-carbonyl)glycinyl oxazolidinone derivative **PH-189** (Figure 1). We hereby report the in vitro antimycobacterial activity of the previously reported *N*-substituted-glycinyl-1*H*-1,2,3-triazolyl oxazolidinones and the development of a fast, reliable, and validated bio-analytical liquid chromatographic–mass spectrometric (LC-MS) method of analysis for estimating the plasma concentration of **PH-189** in the presence of other biological constituents and to indicate any product(s) of instability. Literature survey showed that different instrumental methods have been reported for assessing analysis of structurally diverse oxazolidinone derivatives and their instability products in plasma [16,17,18,19,20].

## 2. Chemistry, Anti-Mycobacterial Susceptibility Testing, and Liquid Chromatography–Mass Spectrometry Instrumentation

The 12 compounds (**PH-145**, **-150**, **-151**, **-165**, **-169**, **-172**, **-181**, **-182**, **-185**, **-189**, **-193**, and **-195**, Table 1) tested in this study were previously reported from our laboratory and were synthesized according to previously reported literature synthetic methods and characterized using appropriate spectroscopic and analytical methods [12]. Antimycobacterial screening assays were performed by National Institute of Health, National Institute of Allergy and Infectious Diseases (NIH/NIAID), USA according to reported methodologies. The minimum inhibitory concentration (MIC, μg/mL) screening was conducted for *Mtb* H37Rv (SRI 1345), isoniazid (INH)-resistant *Mtb* (SRI 1369), rifampin (RMP)-resistant *Mtb* (SRI 1367), and ofloxacin (OFX)-resistant *Mtb* (SRI 4000) strains. The minimum bactericidal concentration (MBC, μg/mL) and low-oxygen recovery assay (LORA, μg/mL) drug screening assays were conducted using only *Mtb* H37Rv (SRI 1345) [21,22,23,24]. Low-oxygen recovery assay was performed to assess the effect of compounds against *Mtb* in a state of non-replicating persistence (NRP). Furthermore, an intracellular (macrophage) drug screening assay was conducted to evaluate the intracellular effectiveness of the compounds [25,26]. Finally, a drug cytotoxicity control plate assay (drug cytotoxicity testing/cell proliferation assay (MTT)) was also performed in parallel to the intracellular drug screening assay using uninfected macrophages to confirm that the concentrations utilized for testing were non-toxic to macrophages. The method validation and analysis of representative compound *N*-(5-nitrofuran-2-carbonyl)glycinyl oxazolidinone derivative (**PH-189**) was performed for the evaluation and quantitation of plasma instability. A Waters Acquity ultra High-Performance Liquid Chromatography (UHPLC) system with quaternary Solvent Manager (H-Class), Sample Manager and UV detector, Waters Acquity UHPLC BEH C18, 1.7 µm, 2.1 × 50 mm analytical column (Waters, Milford, MA, USA) were used for the analysis and method validation. Empower software (Waters) was used for data processing and reporting. Additionally, Waters Xevo G2-S QToF coupled with Waters Acquity UHPLC system with binary Solvent Manager (I-Class) via electrospray ionization (ESI) interface to perform a stress stability study and to identify impurities and degradation pathways. Linearity, precision, accuracy, extraction recovery parameters, and sensitivity; limits of quantitation (LOQ) and detection (LOD) were determined to validate the assay methodology. 

## 3. Results and Discussion

### 3.1. Antimycobacterial Activity

A total of twelve *N*-substituted-glycinyl triazolyl oxazolidinone derivatives were selected and evaluated for antimycobacterial activity against susceptible and resistant *Mtb* strains. Susceptibility testing was performed by the NIH/NIAID, USA according to reported methodologies. The MICs (μM) that would inhibit the growth of *Mtb* H37Rv (SRI 1345), INH-resistant *Mtb* (SRI 1369), RMP-resistant *Mtb* (SRI 1367), and OFX-resistant *Mtb* (SRI 4000) were determined, and the data are presented in Table 1. Overall, all of the compounds showed moderate to potent activity against susceptible and resistant *Mtb*, with MIC values in the ranges 0.5–11.5, 0.056–11.6, 0.11–5.8, and 0.03–11.6 μM, (Table 1) against susceptible (*Mtb* H37Rv), isoniazid-resistant (INH-R), rifampicin-resistant (RMP-R), and ofloxacin-resistant (OFX-R) *Mtb*, respectively. While the percent inhibition for these MIC values ranged between 41–79%, 51–72%, 50–75%, and 52–71%, against *Mtb* H37Rv, INH-R, RMP-R, and OFX-R *Mtb*, respectively. On the contrary, rifampicin showed MIC values of 0.06, 0.06, and 0.95 μM against *Mtb* H37Rv, INH-R, and OFX-R *Mtb*, respectively. The antimycobacterial activity of the compounds was dependent on the substitution at the piperazine 4-*N* position, those containing R’ as H and acyl moieties demonstrated slightly inferior activity in comparison to the aroyl and heteroaroyl substituted derivatives. The presence or absence of a C-4 methyl group on the triazolyl group did not have significant effect on antimycobacterial activity. Furthermore, the nitroaroyl derivatives—namely, the 3,5-dinitrobenzoyl (**PH-181** and **-193**) and the 5-nitrofuroyl (**PH-145** and **-189**) containing derivatives—demonstrated potent antimycobacterial activities with MIC value ranges of 0.052–3.3 and 0.03–0.5 μM, respectively, against all *Mtb* strains tested. Moreover, the 5-nitrofuroyl derivatives (**PH-145** and **-189**) were the most potent and showed excellent activity against susceptible and resistant *Mtb*, with MIC values in the ranges 0.5, 0.056–0.11, 0.11–0.46, and 0.03–0.056 μM (Table 1) against susceptible (*Mtb* H37Rv), INH-R, RMP-R, and OFX-R *Mtb*, respectively. The derivative with unsubstituted triazolyl moiety **PH-189**, demonstrated the most potent activity. However, none of the derivatives evaluated had bactericidal property.

It is known that *Mtb* can reside in a state of NRP representing a state of latent *Mtb* infection. Therefore, LORA (μM) screening assays were conducted using *Mtb* H37Rv (SRI 1345) to assess the effect of compounds against the organism in a state of NRP and reported as the lowest concentration (μM) of compounds that visually inhibited growth of the organism (Table 2). In this assay, only eight of the compounds (namely, **PH-145**, **-169**, **-181**, **-182**, **-185**, **-189**, **-193**, and **-195**) showed activity with MIC values in the range of ≤0.015–5.7 μM, while the other four derivatives (**PH-150**, **-151**, **-165**, and **-172**) were not active with MIC >58 μM, compared to rifampicin with MIC value of 0.95 μM. The activity of the compounds against intracellular (macrophage) *Mtb* was conducted at three different concentrations chosen based on the MIC data generated from the high-throughput screening (HTS) primary screen data, comprising the mid concentration as the MIC, the lower concentration ten-fold below the mid-MIC, and the higher concentration ten-fold above the mid concentration. The intracellular activity data presented as log reduction values calculated as reduction in *Mtb* concentration from zero hour to 7 days post-infection are presented in Table 2. Generally, the log reduction in *Mtb* for most of the compounds with potent activity was within the same range 0.8–2.09 and were comparable to that of rifampicin range of 1.62–2.43 at the three concentrations tested. MTT was also performed at same three concentrations and are reported as percentage cell viability in Table 2. Most of the compounds with potent activity and the reference drug rifampicin used as positive control gave high percent cell viability in the ranges of 73–100% and 84–92%, respectively. 

The *N*-substituted-glycinyl triazolyl oxazolidinone [12] and the *N*-substituted-glycinyl acetamido oxazolidinone [27] derivatives have been reported to demonstrate potent antibacterial activities against susceptible and resistant Gram-positive bacterial strains. In particular, the nitroaroyl and nitroheteroaroyl derivatives have been the most potent against Gram-positive bacteria with extended and superior antibacterial activity against susceptible and clinical isolates of *M. catarrhalis* in comparison to linezolid. The potent antibacterial activities of the nitroaroyl and in particular the 5-nitrofuroyl derivatives made them potential candidates to consider for further antibacterial activity evaluation in vivo in animal infection models. Hence we decided to select a representative example **PH-189** and investigate the development of a fast, reliable, and validated bio-analytical LC-MS method of analysis to estimate its plasma concentration in the presence of other biological constituents and to indicate any product(s) of instability.

### 3.2. Analytical Methods Development and Stability Studies of **PH-189**

#### 3.2.1. Method Development

Different proportions of organic modifiers and buffers with different pH levels were tried in order to obtain optimum resolution, symmetric peak shape, reasonable run time, and optimal sensitivity. Buffers of pH 3, 5, and 7 were investigated, and optimum resolution and peak shape was obtained with 15 mM Tris buffer pH 4 adjusted by 0.01 M HCl/acetonitrile (55:45 *v*/*v*) as a mobile phase. The flow rate for better resolution and rapid separation was adjusted to 0.4 mL/min [supplementary material].

#### 3.2.2. Calibration Curve

Under the developed chromatographic (UHPLC) conditions, relative standard deviation (RSD)% of **PH-189** and internal standard were 0.92% (0.878 ± 0.008) and 1.34% (1.341 ± 0.018), respectively. In addition, the relative peak area of **PH-189**/internal standard and **PH-189** concentrations were linear in the range of 1–90 μg/mL with correlation coefficient (*r*) ≥0.99. From the calibration curve (which was performed in triplicates), the slopes and correlation coefficients showed high consistency, demonstrating the reliability of the standard curve over the concentration ranges studied [supplementary material]. 

#### 3.2.3. Accuracy and Precision

Data for intra- and inter-assay precision and accuracy were derived from the analysis of **PH-189** in human plasma in single day (intra-) and within 10 days (inter-). As shown in Table 3 and Table 4, the intra-assay RSD values were 1.4% to 7.9%, whereas the inter-assay RSDs were 2% to 8.1%. The intra-accuracy was in the range 92.32% to 112.5%, while the inter-accuracy range was 94.41% to 113.4%. From data collected, plasma samples of **PH-189** were stable for at least 12 h at room temperature and for at least 10 days at frozen temp −20 °C. 

#### 3.2.4. Extraction Recovery

The efficiency of the extraction procedure for **PH-189** and internal standard using solid phase extraction (SPE) was assessed by calculating the extraction recovery percentages. The extraction recovery percentages were estimated by comparing the slopes of the calibration curves of extracted and non-extracted sets. The results showed high extraction recoveries of **PH-189** from human plasma with average of 97.44% ± 0.015% (Table 5).

#### 3.2.5. Limit of Quantification and Limit of Detection

LOQ and LOD were calculated by extracting plasma spiked with **PH-189** at concentration 0.1–1 μg/mL. The LOQ was found to be 1 μg/mL. This concentration gave RSD of 7.8% and accuracy of 112.5% of the nominal concentration. However, the LOD for **PH-189** was found to be 0.2 μg/mL using 1 μL as an injection volume.

#### 3.2.6. Evaluation of **PH-189** Stability in Human Plasma

**PH-189** was stable in human plasma and no degradants were detected. The average amount of **PH-189** was calculated from the calibration curve equation and was found to be equal to 48.79 μg/mL and RSD of 0.27%. Thus, the extraction method was able to extract a mean value of 97.44% ± 0.015% of **PH-189** from human plasma (Table 5), which is indicative of high extraction efficiency [supplementary data]. 

#### 3.2.7. Degradation Studies

**Figure 2:**
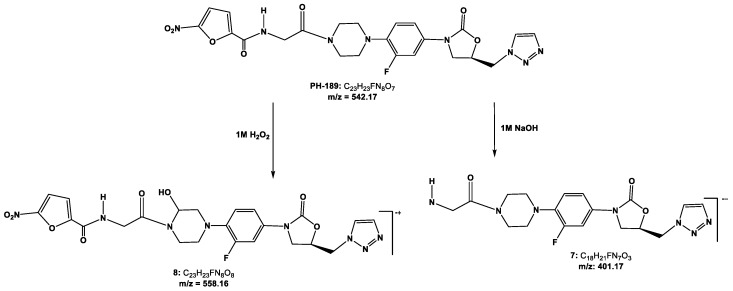
Degradation pathways and degradation products of **PH-189** under basic and oxidative conditions after 90 min storage at 90 °C.

**Figure 3:**
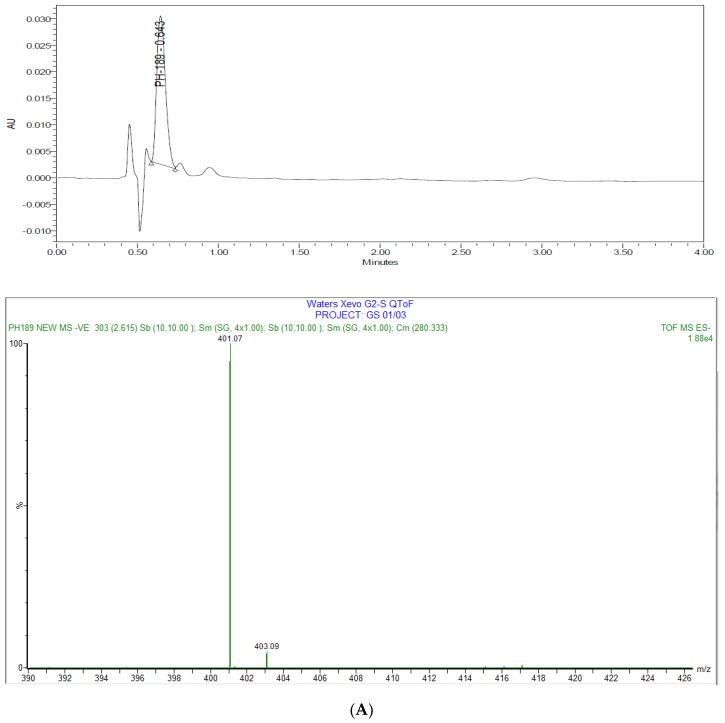

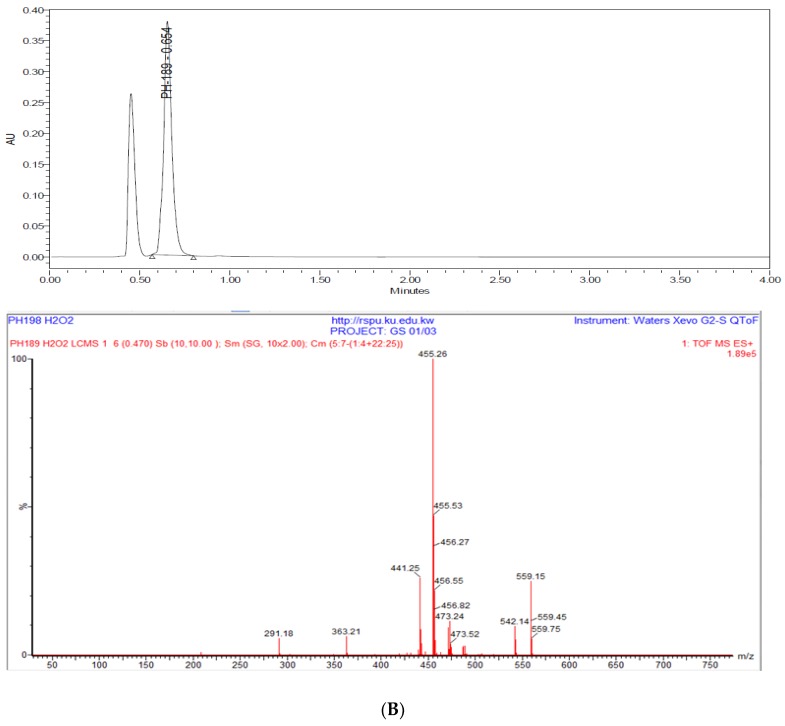
Stability studies under stress conditions UHPLC-Electro Spray Ionisation-Qadrupole Time of Flight Mass Spectrometry (UHPLC-ESI-QToF) analysis under: (**A**) basic condition (1 M NaOH) was operated with negative mode electrospray ionization (ESI) (M^−1^) and (**B**) H_2_O_2_ was operated in positive mode ESI (M + 1).

Degradation pathways and products were identified by UHPLC-Electro Spray Ionisation-Qadrupole Time of Flight Mass Spectrometry (UHPLC-ESI-QToF) as shown in Figure 2. Under acidic condition, it was found that **PH-189** was stable and there were no detectable degradants. However, under basic condition, one small peak was co-eluted with **PH-189** peak as shown in Figure 3A. The peak was due to the hydrolysis of the amide bond resulting in a degradant identified as compound **7**, *N*-methyl-5-nitrofuran-2-carboxamide with *m*/*z* = 401 as shown in Figure 2. Moreover, under oxidative stress condition, using 1 M hydrogen peroxide (H_2_O_2_) at 90 °C for 90 min, the formation of a white precipitate was noticed at the bottom of the vial and precipitate was soluble in water but not in acetonitrile solvent. After dissolving precipitate in a few amount of water the volume was completed with acetonitrile, the sample was injected in UHPLC, and a chromatogram with two prominent peaks was obtained as shown in Figure 3B. After sample analysis by UHPLC-ESI-QToF, **PH-189** was found to be degraded to give a compound corresponding to a peak possessing a *m*/*z* of 558, which was identified as *N*-(2-(4-(4-((*R*)-5-((1*H*-1,2,3-triazol-1-yl)methyl)-2-oxooxazolidin-3-yl)-2-fluorophenyl)-2-hydroxypiperazin-1-yl)-2-oxoethyl)-5-nitrofuran-2-carboxamide resulting from the hydroxylation of the morpholine ring of **PH-189** due to oxidation reaction.

## 4. Conclusions

In conclusion, a series of previously reported *N*-substituted-glycinyl triazolyl oxazolidinone derivatives (12) with potent Gram-positive antibacterial activity and extended activity to selected Gram-negative bacterial strains were evaluated for antimycobacterial effects. Most of the compounds demonstrated moderate to potent antimycobacterial activities against susceptible (*Mtb* H37Rv) and resistant (namely, INH-R, RMP-R, and OFX-R) *Mtb* strains, respectively. Furthermore, representative compounds including the nitroaroyl and nitroheteroaroyl derivatives demonstrated activity against *Mtb* H37Rv (MIC range ≤ 0.008–3 μg/mL) in a state of NRP in the LORA (μg/mL) screen, suggesting their effectiveness against latent *Mtb* infections. None of the derivatives tested showed bactericidal property, but most of them were effective in reducing intracellular (macrophage) *Mtb*. Most of the potent oxazolidinones were found to be non-cytotoxic. A fast, reproducible, and reliable UHPLC-ESI-QToF analytical method was developed for quantitation and stability indication of selected representative compound **PH-189**. This compound was found to be stable under a stress condition in 1 M HCl, but unstable under stress conditions in basic 1 M NaOH and oxidative 1 M hydrogen peroxide solutions, respectively. However, since compound **PH-189** was stable in human plasma with high percent recovery, it is a potent candidate that can be subjected to pharmacological studies in vivo in animal mycobacterial infection models. Previous studies from our laboratory have demonstrated that the MIC value of **PH-189** (calculated partition coefficient, Clog *p* value = 0.5680) remained unchanged in the presence of 50% human plasma against *S. aureus* in vitro, suggesting plasma stability and/or insignificant plasma protein binding [12]. The Clog *p* value was estimated using PerkinElmer, ChemDraw Ultra 13.0 (CambridgeSoft, Waltham, MA, USA, 2012). Therefore, data from this study further emphasizes the potential for **PH-189** as a promising candidate that should be evaluated further. Based on this, further studies are on-going in our laboratories in collaboration with the NIH-NIAID, USA to perform in vivo evaluation and further structural modifications to obtain a more potential drug-like derivative with appropriate pharmacokinetic and pharmacodynamics properties that could be a candidate for further development.

## 5. Materials and Methods

### 5.1. Materials

The 12 compounds screened in this study are previously reported from our laboratory and were synthesized according to literature methods (Ref), and included **PH-145**, **-150**, **-151**, **-165**, **-169**, **-172**, **-181**, **-182**, **-185**, **-189**, **-193**, and **-195** (Table 1). The compounds were purified by silica gel column chromatography and/or recrystallization from suitable organic solvents. Column chromatography was carried out with silica gel (Kieselgel 60, 70–230 mesh; Aldrich, St. Louis, MO, USA) and Thin Layer Chromatography (TLC) conducted on 0.25 mm pre-coated silica gel plates (60F_254_, Merck, Darmstadt, Germany). Compounds were characterized by melting points determined on a Stuart Scientific melting point apparatus (SMP1) (Stuart, Stone, UK) and other spectrometric and CHN analytical methods. The melting points were uncorrected. ^1^H-NMR and ^13^C-NMR spectra were recorded on a Bruker Avance II 600 NMR spectrometer. The chemical shifts of protons are assigned in parts per million (ppm) downfield from tetramethylsilane (TMS) as an internal reference or DMSO-d_6_ (δ = 2.5; 39.7) as solvent. Mass spectra were recorded on a Thermo Scientific DFS High Resolution Gas Chromatography/Mass Spectrometer (DFS GC-MS) (Thermo Scientific, Waltham, MA, USA) and Micro Mass Quattro LC (McKinley, Sparta Township, NJ, USA) Mass Spectrometer. Infrared (IR) spectra were recorded on JASCO FT-IR-6300 (JASCO, Halifax, NS, Canada) spectrometer. Elemental analyses were performed on an Elementar Vario Micro Cube CHN Analyzer apparatus (Elementar, Langenselbold, Germany), and analyses indicated by the symbols of the elements were within ±0.4% of the theoretical values. Analyses were performed at the Science Analytical Facilities (SAF), Faculty of Science, and Department of Pharmaceutical Chemistry, Faculty of Pharmacy, Kuwait University, Kuwait. 

### 5.2. Mycobacterium Tuberculosis Assay

Antimycobacterial testing was performed by NIH/NIAID, USA. *M. tuberculosis* H37Rv (*Mtb* H37Rv; ATCC 27294) was obtained from the American Type Culture Collection (Manassas, VA, USA). Minimum inhibitory concentration (MIC, μM) screening was conducted for *Mtb* H37Rv (SRI 1345), isoniazid (INH)-resistant *Mtb* (SRI 1369), rifampin (RMP)-resistant *Mtb* (SRI 1367), and ofloxacin (OFX)-resistant *Mtb* (SRI 4000). Minimum bactericidal concentration (MBC, μM), low-oxygen recovery assay (LORA, μM), and intracellular (macrophage) drug screening assays were conducted using only *Mtb* H37Rv (SRI 1345). Drug cytotoxicity control plate assay (MTT cell proliferation) was also performed in parallel using uninfected macrophages to confirm that the concentrations utilized for testing were not toxic to the macrophages. Compounds stocks of 10 mM in 100% DMSO were diluted in media and adjusted with DMSO to maintain the final 1% DMSO concentration throughout. 

### 5.3. Minimal Inhibitory Concentration (MIC)

The broth microdilution assay format following guidelines established by the Clinical and Laboratory Standards Institute (CLSI) [22] was utilized for MIC determination. Testing was conducted using 96-well, U-bottom microplates with an assay volume of 0.2 mL/well, as follows. First, the test media, Middlebrook 7H9 broth supplemented with Oleic Albumin Dextrose Catalase (OADC) Enrichment (BD BioSciences; Sparks, MD, USA), was added (0.1 mL/well) to each well. The tested compounds, solubilized in DMSO and subsequently diluted in test media, were added (0.1 mL/well) to appropriate wells at twice the intended starting concentration and serially diluted two-fold across the plate. The plates were then inoculated (0.1 mL/well) with a targeted concentration of 1.0 × 10^6^ CFU/mL *Mtb* and incubated at 37 °C for 7 days in approximately 90% humidity. After incubation, the plates were read visually and individual wells scored for turbidity, as partial clearing or complete clearing. Testing was conducted in duplicate and the following controls were included in each test plate: (i) medium only (sterility control); (ii) organism in medium (negative control); and (iii) rifampin or isoniazid (positive control). The MIC represents the lowest concentration (μM) of drug that visually inhibits growth of the microorganism.

### 5.4. Minimal Bactericidal Concentration

The MBC was determined subsequent to MIC testing by sub-culturing diluted aliquots from wells that fail to exhibit macroscopic growth [22]. The sample aliquots were inoculated onto Middlebrook 7H10 agar plates and subsequently incubated for 16–21 days at 37 °C. Once growth was readily apparent, the bacterial colonies were enumerated. MBC represents the lowest concentration (μM) of compound exhibiting 99.9% kill over the same time period used to determine the MIC (18–24 h). MBC values greater than 16 times the MIC typically indicate antimicrobial tolerance. 

### 5.5. Low-Oxygen Recovery Assay

Traditionally, the screening of compounds against *M. tuberculosis* only addresses or targets the organism in an active replicating state. It is well documented that *Mtb* can reside in a state of NRP, which has not been adequately assessed in the development of new antimicrobials. The microplates were prepared in the same manner as the MIC testing format [21,22,23,24]. However, instead of incubating aerobically, the plates are placed under anaerobic conditions using a modified atmosphere controlled system (MACS) MIC automated jar gassing system and incubated for 7 days at 37 °C. The plates were subsequently transferred to an ambient gaseous condition (5% CO_2_) for 7 days, after which the plates were read visually and individual wells scored for turbidity, as partial clearing or complete clearing. Assay was conducted in duplicate and the following controls were included in each test plate: (i) medium only (sterility control); (ii) organism in medium (negative control); and (iii) rifampin or isoniazid (positive control). The results are reported as the lowest concentration (μM) of drug that visually inhibits growth of the microorganism. 

### 5.6. Intracellular (Macrophage) Drug Screening Assay

Murine J774 cell line was propagated in RPMI 1640 supplemented with l-glutamine and fetal bovine serum (FBS). The cells were maintained in tissue culture flasks at 37 °C in the presence of 5% CO_2_. For infection studies, J774 cells were transferred to 12-well tissue culture chambers in 1 mL volumes at a density of 2.0 × 10^5^ in the presence of 10% FBS [25,26]. After overnight incubation, the medium was replaced with fresh medium containing 1% FBS to stop macrophage division while maintaining cell viability. Twenty-four hours later, the macrophage monolayer was enumerated with an ocular micrometer for total number of cells per well to determine the infection ratio. The medium was removed and replaced with 1 mL of fresh medium with 1% FBS containing *Mtb* at a multiplicity of infection (MOI) of five Mycobacteria/macrophage. The cells were infected for 4 h, after which time nonphagocytosed Mycobacteria were washed from the monolayers and fresh medium was added. Drugs were then added, using three concentrations, and infection was allowed to proceed for 7 days. At 0 and 7 days, the macrophages were lysed with sodium dodecyl sulfate, treated with DNase, diluted, and plated onto 7H10 agar to determine the cell number or colony forming units (CFU). Each drug concentration was tested in duplicate and rifampin was used as the positive control drug. A drug cytotoxicity control plate assay (MTT proliferation) was also conducted in parallel using uninfected macrophages to confirm that concentrations utilized for testing were not toxic to the macrophages. 

### 5.7. Method Development and Validation for Analysis of **PH-189**

#### 5.7.1. Instrumentation and Chromatographic Conditions

A rapid isocratic elution was carried out on a Waters Acquity UHPLC system with quaternary Solvent Manager (H-Class), Sample Manager, and UV detector, Waters Acquity UHPLC BEH C18, 1.7 μm, 2.1 × 50 mm analytical columns were used for the analysis and method validation. The mobile phase comprised filtered and degassed 15 mM Tris buffer in water (pH 4) and acetonitrile in proportion of 55:45 *v*/*v* and pumped at a flow rate of 0.4 mL/min. Column temperature was set to 30 °C and 1 μL of the sample was injected and analyzed at a wavelength of 220 nm. 

#### 5.7.2. Standard Solutions of **PH-189** and Internal Standard

Stock standard solutions of **PH-189** and indapamide as internal standard were separately prepared by dissolving 10 mg of the compounds in 100 mL of acetonitrile to give stock concentrations of 100 μg/mL. Working solutions of **PH-189** and internal standard were prepared by diluting the stock solutions with acetonitrile to obtain concentrations of 10 μg/mL and 90 μg/mL. Stock solutions were stable for at least 5 weeks when stored in a refrigerator (4 °C). 

#### 5.7.3. Human Plasma Extraction Procedure

Aliquots of 0.5 mL of plasma samples (calibration standards) were mixed with 200 μL aliquots of internal standard solution using appropriate concentration (10–90 μg/mL) for **PH-189** concentrations. The samples were loaded unto solid phase extraction (SPE) cartridges on a vacuum 20 position extraction manifold (Waters). The cartridges were pre-conditioned with 1 mL of acetonitrile followed by 1 mL of water. The loaded cartridges were washed with 1 mL of acetonitrile and the analytes (**PH-189** and internal standard) were eluted with acetonitrile into a 1.5 mL glass vials. The collected samples were filtered using Kinesis syringe membrane filters (Kinesis Ltd, St Neots, UK) (13 mm) and were used for injection and analysis. The **PH-189**/internal standard ratios were used to calculate calibration curve and to determine **PH-189** concentration in human plasma after 90 min at 37 °C.

### 5.8. Method Validation Criteria for **PH-189**

#### 5.8.1. Calibration Curve (Linearity)

The linearity of response was estimated using freshly prepared stock solution containing 100 μg/mL of **PH-189**. The linearity was tested for five different concentrations 1, 10, 50, 70, and 90 μg/mL. The final concentration of internal standard was 20 μg/mL. The spiked samples were extracted as described above. Three calibration curves of **PH-189** concentrations were constructed. 

#### 5.8.2. Accuracy and Precision

Accuracy and precision of the UHPLC method for **PH-189** was evaluated by preparing six sets of **PH-189** in blank human plasma within the concentration ranges of the calibration curve. Accuracy and precision were performed in triplicate using four concentration levels of 1, 10, 50, and 90 μg/mL. A set (*n* = 3 of each) was prepared at room temperature (22–25 °C), while five other sets (*n* =3 of each) were prepared and stored at −20 °C (frozen) for 10 days. Freezer-stored samples were thawed, extracted, and analyzed as previously mentioned. Percentage relative standard deviation (%RSD) and percentage deviation from the nominal concentration (% DEV) were used to calculate the intra- and inter-assay precision and accuracy. 

#### 5.8.3. Sensitivity (Limit of Detection and Limit of Quantification)

The LOD and LOQ for **PH-189** were determined at a signal-to-noise ratios of 3:1 and 10:1, respectively. Human plasma samples of **PH-189** were prepared at concentrations of 0.1–1 μg/mL. The methods of extraction using SPE were performed as previously described. 

#### 5.8.4. Extraction Recovery

Two sets of standards containing 1, 50, 70, and 90 μg/mL of **PH-189** were prepared. One set was prepared in human plasma and the other set in the mobile phase. The plasma standards were mixed with 20 μg of internal standard and extracted as mentioned previously, while the standards of the other set were directly injected after mixing with internal standard (non-extracted samples). The extraction recoveries were calculated from the slopes of the standard curve of **PH-189** in plasma and mobile phase. Absolute recoveries of **PH-189** and internal standard were also determined by comparing the absolute values of the peak areas of **PH-189** and internal standard in extracted and non-extracted samples.

#### 5.8.5. Evaluation of **PH-189** Stability in Human Plasma

Three samples containing 50 μg/mL of **PH-189** and 20 μg/mL of internal standard were spiked in human plasma and placed in an oven for 90 min at 37 °C. **PH-189** was extracted from plasma by using solid phase extraction cartridges (Sep-Pak Vac C18) (Waters, Milford, MA, USA) and filtered by syringe membrane filters (13 mm) Kinesis. 

#### 5.8.6. Degradation Studies

For degradation studies, three 4 mL vials containing 5 mg of **PH-189** dissolved in 5 mL acetonitrile and 5 mL of 1 M of HCl, 1 M of NaOH, and 1 M of H_2_O_2_ was added to each vial. The samples were heated to 90 °C for 90 min and evaporated using nitrogen gas. Then, 1 mL of acetonitrile was added while scratching the vial’s inner walls after allowing the samples to cool down for 15 min. Finally, 2 μL solutions were injected for analysis and the presence of interfering peak(s) eluted at/or near the retention time of **PH-189** was checked for degradants that were identified by UHPLC–ESI-QToF. All determinations were conducted in triplicates. 

## Figures and Tables

**Table 1:**
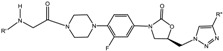
Antimycobacterial activity (minimum inhibitory concentration, minimum inhibitory concentration (MIC), and minimum bactericidal concentration (MBC, μM)) of *N*-substituted glycinyl triazolyl oxazolidinones.

Compound Code	R′	R″	MIC H37Rv	% Inhibition ^a^	MBC H37Rv	MIC INH-R ^b^	% Inhibition	MIC RMP-R ^c^	% Inhibition	MIC OFX-R ^d^	% Inhibition
**PH-172**	H . CF_3_CO_2_H	H	3.6	59	N/A ^e^	7.2	68	3.6	63	3.6	62
**PH-151**	H . CF_3_CO_2_H	CH_3_	5.8	63	N/A	11.6	68	5.8	73	11.6	71
**PH-165**	CH_3_CO	H	6.7	62	N/A	4.5	68	6.7	70	3.4	63
**PH-182**	CHCl_2_CO	CH_3_	1.9	75	N/A	0.95	67	0.95	67	0.47	68
**PH-169**	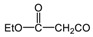	H	2.9	51	N/A	1.45–2.9	53–66	1.45	61	1.45	69
**PH-150**	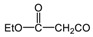	CH_3_	5.8	79	N/A	2.9	59	2.9	66	2.9	69
**PH-193**	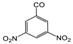	H	1.7	68	N/A	0.42	59	0.22	66	0.052	52
**PH-181**	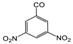	CH_3_	3.3	76	N/A	0.85–1.64	51–63	0.82	60	0.82	66
**PH-189**	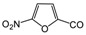	H	0.5	57	N/A	0.057–0.12	59–65	0.46	58	0.03	56
**PH-145**	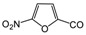	CH_3_	0.5	58	N/A	0.056–0.11	51–64	0.11	50	0.056	65
**PH-195**	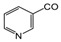	H	3.9	71	N/A	2	58	0.98	75	0.49	63
**PH-185**	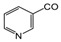	CH_3_	5.7–11.5	41–68	N/A	2.9	72	5.7	63	1.44	60
RMP ^f^	-	-	0.06	66	1.56	0.06	58	N/A ^g^	N/A	0.95	56
INH ^f^	-	-	N/A	N/A	N/A	N/A	N/A	0.15	62	N/A	N/A

^a^ Percent inhibition at MIC concentration. ^b^ INH-R = Isoniazid resistance. ^c^ RMP-R = Rifampicin resistance. ^d^ OFX-R = Ofloxacin resistance. ^e^ N/A = Not applicable: Colony counts above the established rejection value of ≥40. ^f^ Rifampicin (RMP) and Isoniazid (INH) are used as positive controls, respectively. ^g^ N/A = Not applicable: Compound not used in this assay.

**Table 2:** Low-oxygen recovery assay (LORA), macrophage, and drug cytotoxicity testing (MTT) data for *N*-substituted glycinyl triazolyl oxazolidinones.

Compound Code	LORA (μM)	Macrophage Log Reduction (Low Conc.)	Macrophage Log Reduction (Mid Conc.)	Macrophage Log Reduction (High Conc.)	MTT % Viability (Low Conc.)	MTT % Viability (Mid Conc.)	MTT % Viability (High Conc.)
**PH-172**	>58	1.76 (0.36)	5.55 (3.6)	1.18 (36)	87	72	71
**PH-151**	>46	1.28 (0.39)	1.06 (3.9)	1.34 (39)	83	87	74
**PH-165**	>54	1.82 (1.1)	1.30 (3.4)	1.13 (34)	91	87	84
**PH-182**	≤0.015	1.72 (0.09)	1.35 (0.95)	1.54 (9.5)	84	90	87
**PH-169**	≤0.091	1.88 (0.39	1.34 (3.9)	1.24 (39)	76	75	73
**PH-150**	>47	1.85 (0.39)	1.06 (3.9)	0.89 (39)	78	82	62
**PH-193**	≤0.11	1.13 (0.17)	1.30 (1.7)	1.78 (17)	100	100	74
**PH-181**	≤0.10	1.78 (0.33)	1.28 (3.3)	2.09 (33)	91	88	39
**PH-189**	0.92	1.81 (0.18)	1.45 (1.8)	1.34 (18)	98	88	86
**PH-145**	0.45	1.41 (0.036)	1.17 (0.36)	1.23 (3.6)	87	93	75
**PH-195**	2	1.06 (0.20)	1.35 (2.0)	1.36 (20)	100	100	71
**PH-185**	5.7	1.48 (0.38)	1.09 (3.8)	0.89 (38)	88	94	39
RMP ^a^	0.95	1.62 (0.12)	2.17 (1.2)	2.43 (12)	92	85	84

^a^ RMP used as positive control.

**Table 3:** Intra-assay precision and accuracy data for **PH-189** determination in human plasma using ultra High-Performance Liquid Chromatography (UHPLC)-UV.

Nominal/μg/mL	Mean ± s (*n* = 3) Observed/μg/mL	Precision ^a^ (%)	Accuracy ^b^ (%)
1	1.125 ± 0.089	7.9	112.5
50	46.16 ± 0.665	1.4	92.32
70	66.28 ± 1.794	2.7	94.69
90	85.67 ± 2.657	3.1	95.19

^a^ Expressed as the relative standard deviation (RSD). ^b^ Expressed as (mean % deviation = mean calculated concentration/nominal concentration X100).

**Table 4:** Inter-assay precision and accuracy data for **PH-189** determination in human plasma using UHPLC-UV.

Nominal/μg/mL	Mean ± s (*n* = 3) Observed/μg/mL	Precision ^a^ (%)	Accuracy ^b^ (%)
1	1.134 ± 0.091	8.1	113.4
50	47.50 ± 0.954	2.0	95.00
70	66.94 ± 2.404	3.6	95.62
90	84.97 ± 3.460	4.1	94.41

^a^ Expressed as RSD. ^b^ Expressed as (mean % deviation = mean calculated concentration/nominal concentration X100).

**Table 5:** Extraction recovery of **PH-189** from human plasma in triplicate using UHPLC-UV.

N (Concentration Range 1–90 μg/mL)	Extracted	Non-Extracted	Recovery (%)
1	0.0421	0.0428	98.36%
2	0.0454	0.0462	98.26%
3	0.0444	0.0464	95.69%
Mean	0.0440	0.0451	97.44%

## Supplementary Materials

The following are available online at www.mdpi.com/2218-0532/85/4/34/s1, Figure S1: Ultra-performance liquid chromatography analysis of **PH-189** and internal standard (indapamide), Figure S2: Calibration curve obtained for relative peak areas of **PH-189** (**PH-189** peak area/ internal standard peak area) versus **PH-189** concentrations, Figure S3: Chromatogram obtained after placing the spiked **PH-189** and internal standard (10 μg/ml and 50 μg/ml, respectively) in human plasma for 90 minutes at 37 °C.

## References

[1] (2016). WHO Global Tuberculosis Report 2016. http://apps.who.int/iris/bitstream/10665/250441/1/9789241565394-eng.pdf?ua=1.

[2] Poce G., Cocozza M., Consalvi S., Biava M. (2014). SAR analysis of new anti-TB drugs currently in pre-clinical and clinical development. Eur. J. Med. Chem..

[3] Brigden G., Hewison C., Varaine F. (2015). New developments in the treatment of drug-resistant tuberculosis: Clinical utility of bedaquiline and delamanid. Infect. Drug Resist..

[4] Szumowski J.D., Lynch J.B. (2015). Profile of delamanid for the treatment of multidrug-resistant tuberculosis. Drug Des. Dev. Ther..

[5] Butler M.S., Blaskovich M.A., Cooper M.A. (2017). Antibiotics in the clinical pipeline at the end of 2015. J. Antibiot..

[6] Fox G.J., Menzies D. (2013). A Review of the Evidence for Using Bedaquiline (TMC207) to Treat Multi-Drug Resistant Tuberculosis. Infect. Dis. Ther..

[7] Wilcox M.H. (2005). Update on linezolid: The first oxazolidinone antibiotic. Expert Opin. Pharmacother..

[8] Butler M.S., Blaskovich M.A., Cooper M.A. (2013). Antibiotics in the clinical pipeline in 2013. J. Antibiot..

[9] Rose P.C., Hallbauer U.M., Seddon J.A., Hesseling A.C., Schaaf H.S. (2012). Linezolid-containing regimens for the treatment of drug-resistant tuberculosis in South African children. Int. J. Tuberc. Lung Dis..

[10] Xu H.B., Jiang R.H., Li L., Xiao H.P. (2012). Linezolid in the treatment of MDR-TB: A retrospective clinical study. Int. J. Tuberc. Lung Dis..

[11] Ager S., Gould K. (2012). Clinical update on linezolid in the treatment of Gram-positive bacterial infections. Infect. Drug Resist..

[12] Phillips O.A., Udo E.E., Abdel-Hamid M.E., Varghese R. (2013). Synthesis and antibacterial activities of *N*-substituted-glycinyl 1*H*-1,2,3-Triazolyl oxazolidinones. Eur. J. Med. Chem..

[13] Phillips O.A., Udo E.E., Abdel-Hamid M.E., Varghese R. (2009). Synthesis and antibacterial activity of novel 5-(4-Methyl-1*H*-1,2,3-triazole) methyl oxazolidinones. Eur. J. Med. Chem..

[14] Phillips O.A., Udo E.E., Ali A.A., Al-Hassawi N. (2003). Synthesis and antibacterial activity of 5-substituted oxazolidinones. Bioorg. Med. Chem..

[15] Phillips O.A., Udo E.E., Varghese R. (2012). Antimycobacterial Activities of Novel 5-(1*H*-1,2,3-Triazolyl)Methyl Oxazolidinones. Tuberc. Res. Treat..

[16] Bae S.K., Kim E.J., Kwon J.W., Kim W.B., Lee M.G. (2003). High-performance liquid chromatographic analysis of DA-7867, a new oxazolidinone, in human plasma and urine and in rat tissue homogenates. J. Chromatogr. B Anal. Technol. Biomed. Life Sci..

[17] Cavazos-Rocha N., Carmona-Alvarado I., Vera-Cabrera L., Waksman-de-Torres N., Salazar-Cavazos Mde L. (2014). HPLC method for the simultaneous analysis of fluoroquinolones and oxazolidinones in plasma. J. Chromatogr. Sci..

[18] Hedaya M.A., Thomas V., Abdel-Hamid M.E., Kehinde E.O., Phillips O.A. (2017). A validated UPLC-MS/MS method for the analysis of linezolid and a novel oxazolidinone derivative (PH027) in plasma and its application to tissue distribution study in rabbits. J. Chromatogr. B Anal. Technol. Biomed. Life Sci..

[19] Phillips O.A., Abdel-Hamid M.E. (2008). Determination of novel antibacterial triazolylmethyl oxazolidinones concentrations in human plasma by APCI-LC-MS: Application to stability study. J. Pharm. Pharm. Sci..

[20] Phillips O.A., Sharaf L.H., Abdel-Hamid M.E., Varghese R. (2011). Assessment of the stability of novel antibacterial triazolyl oxazolidinones using a stability-indicating high-performance liquid chromatography method. Med. Princ. Pract..

[21] Cho S.H., Warit S., Wan B., Hwang C.H., Pauli G.F., Franzblau S.G. (2007). Low-oxygen-recovery assay for high-throughput screening of compounds against nonreplicating *Mycobacterium tuberculosis*. Antimicrob. Agents Chemother..

[22] Clinical and Laboratory Standards Institute (CLSI) (2011). Susceptibility Testing of Mycobacteria, Nocaridae, and Other Aerobic Actinomycetes. Approved Standard.

[23] Cooksey R.C., Crawford J.T., Jacobs W.R., Shinnick T.M. (1993). A rapid method for screening antimicrobial agents for activities against a strain of *Mycobacterium tuberculosis* expressing firefly luciferase. Antimicrob. Agents Chemother..

[24] Wayne L.G., Hayes L.G. (1996). An In Vitro model for sequential study of shiftdown of *Mycobacterium tuberculosis* through two stages of nonreplicating persistence. Infect. Immun..

[25] Barrow E.L., Quenelle D.C., Suling W.J., Barrow W.W. (1996). Use of mono Mac 6 Human Moncytic Cell Line and J774 Murine Macrophage Cell Line in Parallel Antimycobacterial Drug Studies. Antimicrob. Agents Chemother..

[26] Rastogi N., Labrousse V., Goh K.S., De Sousa J.P. (1991). Antimycobacterial spectrum of sparfloxacin and its activities alone and in association with other drugs against *Mycobacterium avium* complex growing extracellularly and intracellularly in murine and human macrophages. Antimicrob. Agents Chemother..

[27] Wang X.J., Wu N., Du G.J., Zhao S.Q., Yan M., Gu L.Q. (2009). Synthesis and antibacterial activities of eperezolid analogs with glycinyl substitutions. Arch. Pharm..

