# Supplementary material for: Antimycobacterial Activities of N-Substituted-Glycinyl 1H-1,2,3-Triazolyl Oxazolidinones and Analytical Method Development and Validation for a Representative Compound
Source: Sci Pharm. 2017 Oct 2;85(4):34. doi: 10.3390/scipharm85040034 (PMC5748531; doi:10.3390/scipharm85040034)
Supplement: Supplementary file 1 [file scipharm-85-00034-s001.docx]

Supplementary Materials


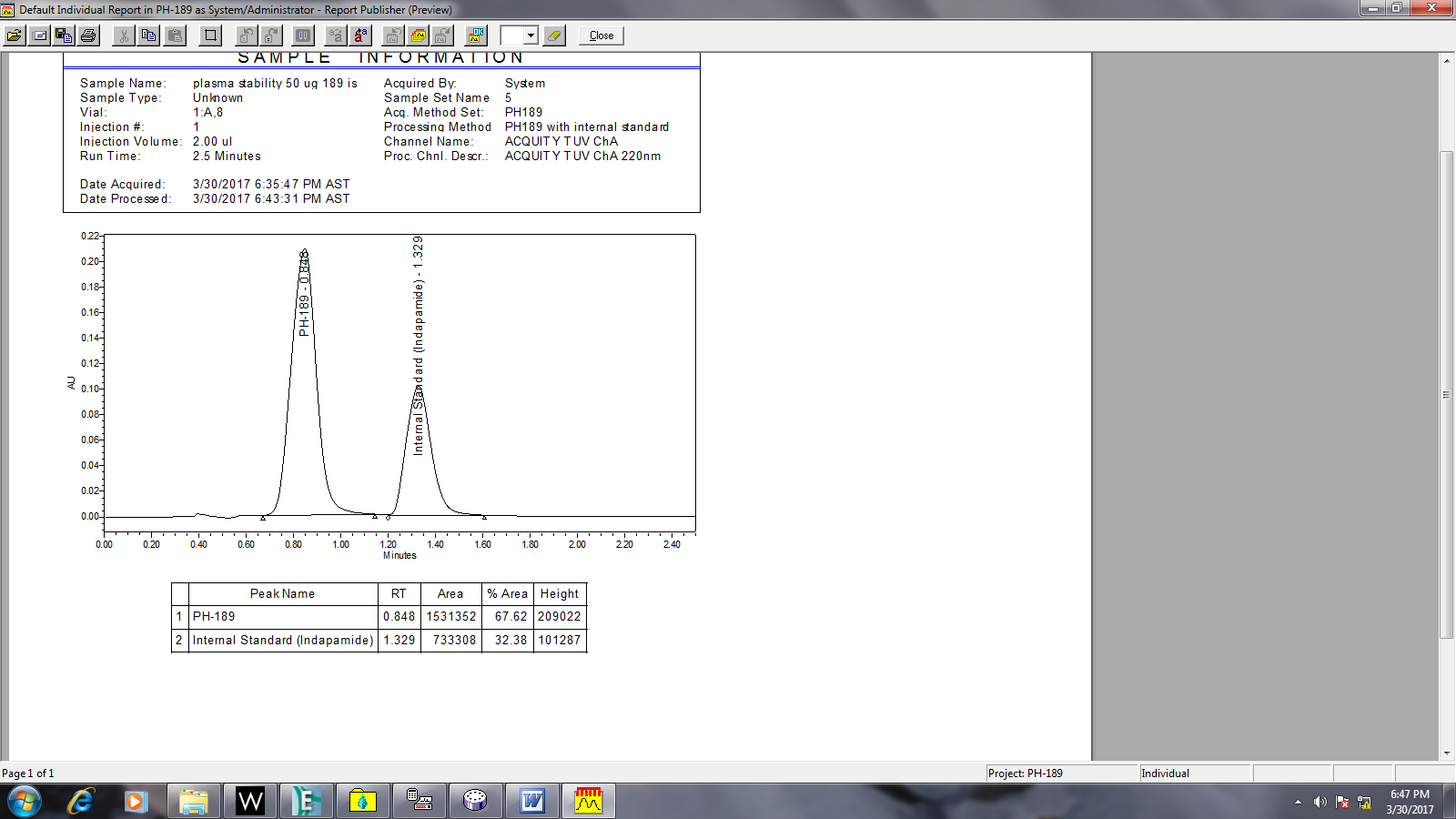


**Figure 1.Ultra-performance liquid chromatography analysis of PH-189 and internal standard (indapamide).**

**Figure 2. Calibration curve obtained for relative peak areas of PH-189 (PH-189 peak area/ internal standard peak area) versus PH-189 concentrations.**


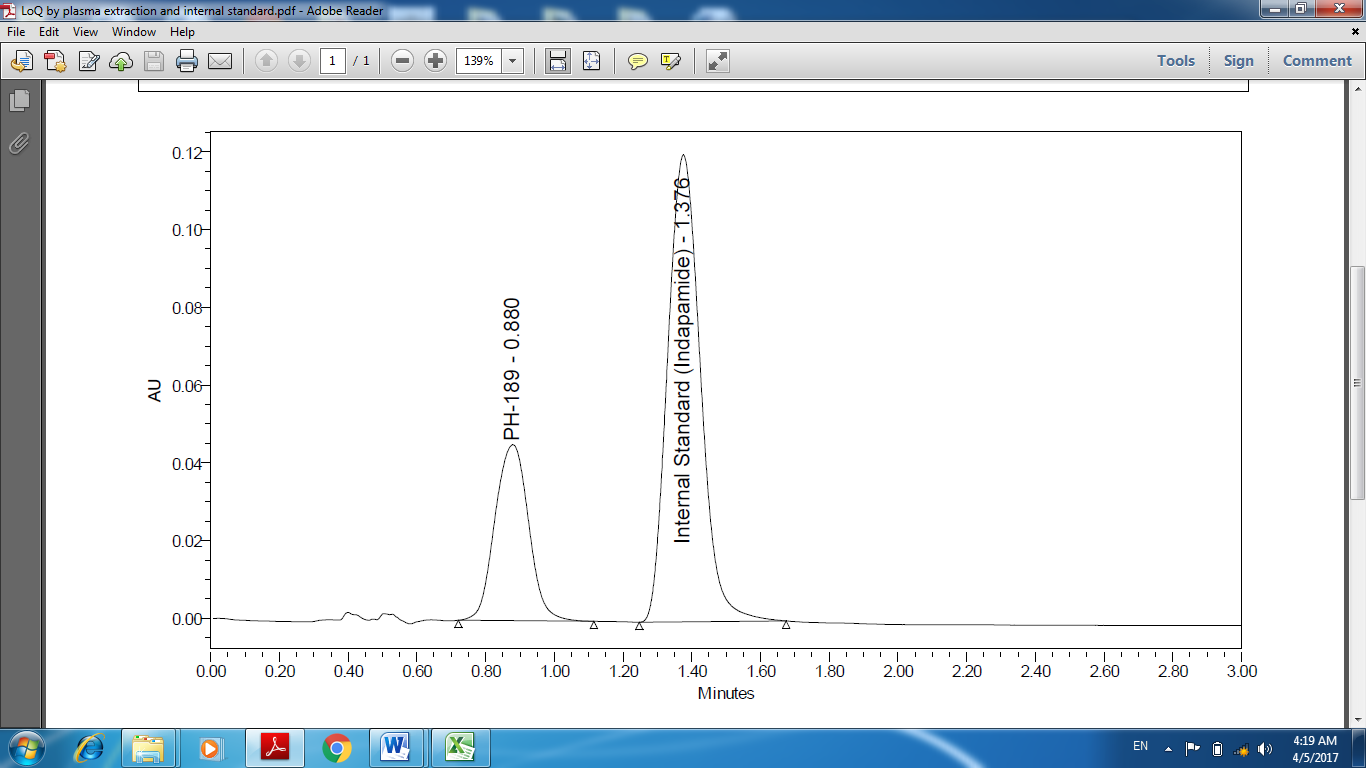


**Figure 3. Chromatogram obtained after placing the spiked PH-189 and internal standard (10 μg/ml and 50** **μg/ml, respectively) in human plasma for 90 minutes at 37 °C.**
